# ADRV 12L: A Ranaviral Putative Rad2 Family Protein Involved in DNA Recombination and Repair

**DOI:** 10.3390/v14050908

**Published:** 2022-04-27

**Authors:** Fei Ke, Qi-Ya Zhang

**Affiliations:** 1 Institute of Hydrobiology, The Innovation Academy of Seed Design, Chinese Academy of Sciences, Wuhan 430072, China; zhangqy@ihb.ac.cn; 2 College of Modern Agriculture Sciences, University of Chinese Academy of Sciences, Beijing 100049, China

**Keywords:** *Andrias davidianus* ranavirus (ADRV), iridoviruses, Rad2, homologous recombination, double-strand break repair

## Abstract

The *Andrias davidianus* ranavirus (ADRV) is a member of the family *Iridoviridae* and belongs to the nucleocytoplasmic large DNA viruses. Based on genomic analysis, an ADRV-encoding protein, ADRV *12L*, and its homologs from other iridoviruses were predicted as Rad2 family proteins based on the conserved amino acids, domains, and secondary structures. Expression analysis showed that the transcription of ADRV *12L* started at 4 h post infection, and its expression was not inhibited by a DNA-replication inhibitor. Meanwhile, immunofluorescence localization showed that ADRV *12L* mainly localized in viral factories and colocalized with the viral nascent DNA, which hinted at a possible role in DNA replication. Furthermore, a mutant ADRV lacking *12L* (ADRV-Δ12L) was constructed. In both luciferase assays based on homologous recombination (HR) and double-strand break repair (DSBR) that followed, ADRV-Δ12L induced less luciferase activity than the wild-type ADRV, indicating that HR and DSBR were impaired in ADRV-Δ12L infected cells. In addition, infection with ADRV-Δ12L resulted in smaller plaque sizes and lower viral titers than that with wild-type ADRV, indicating an important role for *12L* in efficient virus infection. Therefore, the results suggest that Rad2 homologs encoded by iridovirus have important roles in HR- and DSBR-process of the viral DNA and, thus, affect virus replication and the production of progeny virions.

## 1. Introduction

Viruses in the genus *Ranavirus* (family *Iridoviridae*) have large icosahedral capsids and double-stranded DNA genomes, and they belong to the nucleocytoplasmic large DNA viruses (NCLDVs) [1]. Ranaviruses infect ectothermic vertebrates and have been isolated from reptiles, amphibians, and fish [2,3,4]. Due to their wide host range, ranaviruses have caused huge losses in the aquaculture industry and also threaten wild animals [5,6,7]. Although several genomes of ranavirus isolates have been sequenced, information on the functions of their coding proteins is still lacking.

*Andrias davidianus* ranavirus (ADRV) was isolated from the Chinese giant salamander *Andrias davidianus*, which is the largest amphibian in the world [8]. ADRV has a genome of 106.7 kbp possessing 101 potential open reading frames (ORFs). Sequence analysis showed that 26 ORFs of ADRV belonged to the iridovirus core genes, which were conserved in all the sequenced iridoviruses. Among them, the ADRV *12L* ORF was predicted to encode a protein of the Rad2 family.

The Rad2 family of proteins are a large group of structure-specific nucleases that function in response to aberrant nucleic acid structures [9]. As a family of evolutionarily conserved proteins, the nucleases have different names in different organisms, such as Rad2 in *Saccharomyces cerevisiae* [10], and flap endonuclease 1 (FEN1) and XPG in humans [11]. During DNA metabolism, these nucleases are required to repair DNA damage caused during replication or recombination, to maintain genome stability [12,13]. For example, FEN1 functioned to remove the protruding overhangs generated in strand displacement synthesis during lagging strand synthesis in DNA replication [12] and also participates in the treatment of these structures in DNA repair and recombination [14,15]. Due to their importance, this family of proteins have been found in species ranging from higher to lower organisms, even in viruses [16]. The Rad2 homolog has been predicted as a conserved protein in iridoviruses, but its functions are unknown.

The Rad2 homologous proteins in iridoviruses include ADRV *12L*, 95R of frog virus 3 [17], 102R of *Rana grylio* virus [18], *12L* of common midwife toad virus [19], etc. Although the Rad2 homologs are conserved in iridoviruses, they have different sequence identities (ranged from 31% to 99% in vertebrate iridoviruses). However, they all contained the conserved domains/motifs of Rad2 family as revealed by BLAST search, indicating that these proteins might function in virus replication as a Rad2 protein.

As large DNA viruses, ranaviruses have been predicted to encode several enzymes involved in DNA replication and transcription, such as the DNA polymerase, RNA polymerase subunits, and some accessory proteins. In previous work, we screened the proteins involved in virus replication and transcription in ADRV infected cells, in which ADRV *12L* was found [20]. In the present study, we cloned and analyzed the function of ADRV *12L* by subcellular localization, knockout mutant virus, and homologous recombination (HR) assays.

## 2. Materials and Methods

### 2.1. Viruses and Cells

ADRV isolated in our lab was used in this study [8]. A Chinese giant salamander thymus cell (GSTC) line that was established in our lab [21], and an *Epithelioma Papulosum Cyprini* (EPC) cell line that was maintained in our lab [22], were cultured in M199 medium supplemented with 10% bovine calf serum at 25 °C until use.

### 2.2. Sequence Analysis

The nucleotide sequence of ADRV *12L* was extracted from the ADRV genome sequence (GenBank: KC865735.1) and analyzed with the DNASTAR software. Homologous sequences were searched using National Center for Biotechnology Information (NCBI) BLAST. Multiple sequence alignments were constructed using CLUSTAL 1.83 and edited using GeneDoc 2.7. Conserved domains or motifs were searched for in the Conserved Domain Database (CDD, NCBI) or using SMART (http://smart.embl-heidelberg.de/, accessed on 4 August 2021). The structure of the *12L* protein was predicted using RoseTTAFold from the Robetta service (http://robetta.bakerlab.org/, accessed on 18 August 2021) [23].

### 2.3. Antibody Preparation

The coding region for aa 75–240 of ADRV *12L* was amplified by PCR and ligated into the pET32a vector. The recombinant plasmid was used to transform into *Escherichia coli* BL21 (DE3). Positive clones were cultured in LB medium and induced with 1 mM isopropyl-β-D-thiogalactopyranoside (IPTG) for 4 h at 37 °C. The recombinant protein was purified using the HisBind Purification Kit (Novagen, Madison, WI, USA) according to the manufacturer’s instructions. The purified protein was used to immunize mice by intraperitoneal injection. After the fifth immunization, anti-ADRV *12L* serum was collected.

This experiment was carried out in strict accordance with the recommendations in the Regulations for the Administration of Affairs Concerning Experimental Animals of China. The animal procedure and protocol were approved by the Institutional Animal Care and Use Committee of the Institute of Hydrobiology, Chinese Academy of Sciences (Approval number: Y911030401). All efforts were made to minimize suffering.

### 2.4. RT-PCR and Western Blot Analysis

GSTC cells were infected or mock infected with ADRV at an MOI of 0.1 and collected at various times post infection (0, 2, 4, 6, 8, 12, 24, and 48 h). The total RNA was isolated from the samples and subjected to RT-PCR, as described previously [24]. The primers *12L*-F/R (5′-GTGCGGCACAGACTTTAACC-3′/5′-ATGTCCCCGCCAGAGAGTAT-3′) were used to detect the transcription of ADRV *12L*, and *β-actin* was used as a control [25]. 

Western blot analysis was performed with the same samples as described above. Anti-ADRV *12L* serum or anti-β-actin antibody (ABclonal, Wuhan, China) was used as the primary antibody at a dilution of 1:500, followed by horseradish-peroxidase-conjugated goat anti-mouse IgG (ABclonal, Wuhan, China) at a dilution of 1:1000 as the secondary antibody. The detection of the expression of β-actin was used as the internal control. Antibody binding was detected by chemiluminescence (Millipore, Boston, MA, USA).

### 2.5. Cytarabine Treatment

Cytarabine (cytosine β-D-arabinofuranoside, Ara-C), a DNA replication inhibitor, was used to classify the transcription class of ADRV *12L* as described previously [26]. Ara-C was added to GSTC cells at a final concentration of 100 μg/mL for 1 h prior to virus infection. The pre-treated or untreated cells were then infected with ADRV at an MOI of 0.5. The total protein was collected at 0, 24, and 48 h post infection (hpi) for Western blot analysis as described above. In addition to the anti-ADRV *12L* serum, previously prepared anti-ADRV 85L and anti-ADRV MCP serum [20,27] were used as controls.

### 2.6. Immunofluorescence Microscopy

GSTC cells were seeded on coverslips in a 12-well plate and mock infected or infected with ADRV at an MOI of 0.5. For EdU labeling, EdU (Invitrogen, Carlsbad, CA, USA) was added to a final concentration of 10 μM at the indicated times. After a continued incubation for 30 min, the cells were washed with phosphate-buffered saline (PBS) and fixed with 4% paraformaldehyde for 20 min, and then processed with a Click-iT Plus EdU Cell Proliferation Kit for Imaging (Thermo Fisher, Waltham, USA) according to the manufacturer’s protocols as described previously [20]. For antibody staining, EdU labeled or unlabeled cells were incubated with anti-ADRV *12L* or anti-ADRV 85L serum at a dilution of 1:100 as the primary antibody. After washing with PBS containing 1% BSA, the cells were incubated with secondary antibody (Alexa Fluor 546 conjugated goat anti-mouse IgG, Alexa Fluor 488 conjugated goat anti-mouse IgG, or Alexa Fluor 546 conjugated goat anti-Rabbit IgG) at a dilution of 1:1000. Hoechst 33342 was used to stain the cell nucleus. The samples were examined under a Leica TCS SP8 confocal microscope.

### 2.7. Generation of 12L Knockout Virus

DNA fragments of the ADRV genome region before the initiation codon of ADRV *12L* (12L_-L_) and after the stop codon (12L_-R_) were amplified by PCR, using ADRV genomic DNA as a template. The sequence for the selection marker P50-GFP in which the expression of GFP was driven by a ranavirus late promoter was amplified in a previously constructed plasmid [28]. The three fragments were inserted into the pMD18-T vector using the infusion cloning strategy to obtain the recombinant plasmid pMD18T-12L_-L_-GFP-P50-12L_-R_. The recombinant fragment was confirmed by DNA sequencing.

To obtain the 12L-knockout virus, the plasmid pMD18T-12L_-L_-GFP-P50-12L_-R_ was transfected into GSTC cells with Lipofectamine 3000 (Thermo Fisher, Waltham, MA, USA). Six hours after transfection, the cells were infected with ADRV at an MOI of 1 and collected at 48 hpi. The collected cells were subjected to a freeze–thaw cycle for three times, diluted and used to inoculate GSTC cells that had been pre-seeded in a 24-well plate, which were then covered with 0.7% agarose. After 3 days of incubation, the viral plaques emitting green fluorescence were picked and used for another round of infection. After several rounds of infection and picking, a purified virus, ADRV-Δ12L, was obtained, and verified by PCR and Western blotting.

### 2.8. Plaque Assay

GSTC cells seeded in 24-well plate were infected with ADRV or ADRV-Δ12L at a 0.01 MOI. Unbound virus was removed after 1 h of adsorption. The cells were overlaid with medium containing 0.7% agarose and cultured for 3 days. After the plaques formed, the cells were fixed with 20% formaldehyde and stained with 1% crystal violet.

### 2.9. One-Step Virus Growth Curves

GSTC cells were infected with ADRV or ADRV-Δ12L at a 0.1 MOI, and were harvested at various intervals (0, 4, 8, 12, 24, 36, 48, and 72 h). The virus titers were determined on triplicate monolayers of GSTC cells using TCID_50_ assays.

### 2.10. Luc-HR Assay

If ADRV *12L* is a member of the Rad2 family, it could have a function in DNA recombination and repair. Therefore, a luciferase-based homologous recombination (Luc-HR) assay and double strand break repair (DSBR) assay were performed to confirm the speculation. The Luc-HR assay was performed as described with modifications [29]. A plasmid that contained a firefly luciferase encoding gene driven by the promoter of the ADRV *ICP18* gene (immediate-early gene) was constructed. First, the coding region for the firefly luciferase was amplified by PCR using the pGL3-basic vector as a template. The promoter region (P18) of the ADRV *ICP18* gene was amplified using ADRV genomic DNA as a template. The region for the SV40 polyadenylation signal was amplified from the pcDNA3.1 vector and used as a termination signal (T_SV40_). The three fragments were fused by overlap PCR and ligated into the pMD18-T vector to obtain the plasmid P_18_-lucT_(1–2103)_, in which the fragment P18-luciferase-T_SV40_ has a length of 2103 bp. Then, the other plasmids, P_18_-luc_(1–1334)_, lucT_(734–2103)_, lucT_(934–2103)_, and lucT_(1134–2103)_, containing different regions of the P_18_-lucT_(1–2103)_, were constructed. In addition, the coding sequence of the *Renilla* luciferase gene was amplified by using the pRL-TK vector as a template, and then ligated into the plasmid P_18_-lucT_(1–2103)_ to replace the firefly luciferase gene, and the plasmid P_18_-Rluc was obtained. All the plasmids were confirmed by DNA sequencing.

The Luc-HR assay was performed in EPC cells because they allow higher transfection efficiency than GSTC. Before transfection, EPC cells were seeded into 24-well plates and infected with ADRV or ADRV-Δ12L at 0.5 MOI, respectively. After 6 hpi, the cells were transfected with combination of plasmids, P_18_-luc_(1–1334)_ plus lucT_(734–2103)_ or lucT_(934–2103)_ or lucT_(1134–2103)_, respectively. These plasmids were also cotransfected with the pUC19 vector to evaluate the luciferase activity caused by them alone. In these transfections, the plasmid P_18_-Rluc was co-transfected as an internal control. Then, 24 h post transfection (hpt), the cells were collected and the luciferase activity of each well was determined by using the dual-luciferase reporter assay system (Promega, Madison, WI, USA). The firefly luciferase activities obtained in each group were first normalized to the *Renilla* luciferase activity in the group. Then, the firefly luciferase activity was compared with each other.

### 2.11. DSBR Assay

To perform the DSBR assay, two linearized DNA fragments were obtained by PCR amplification using the plasmid P18-lucT_(1–2103)_ as a template. The first was amplified from the ATG start site of the firefly luciferase gene to create a double-strand break between the P18 promoter and the firefly luciferase gene, which was termed DSB1. The other was amplified from the C-terminal of the luciferase gene to obtain a fragment containing C-terminal deleted luciferase gene, which was termed DSB2. These linearized plasmids were produced using Pfu Taq to obtain a blunt end. The two linearized plasmids were cotransfected or each with pUC19 into the virus-infected EPC cells, as described above. The plasmid P_18_-Rluc was also co-transfected as an internal control. The luciferase activities were determined at 24 hpt, as described above. All the primers used in the study are summarized in Supplementary Table S1.

## 3. Results

### 3.1. Sequence Characteristics of ADRV 12L

**Figure 1:**
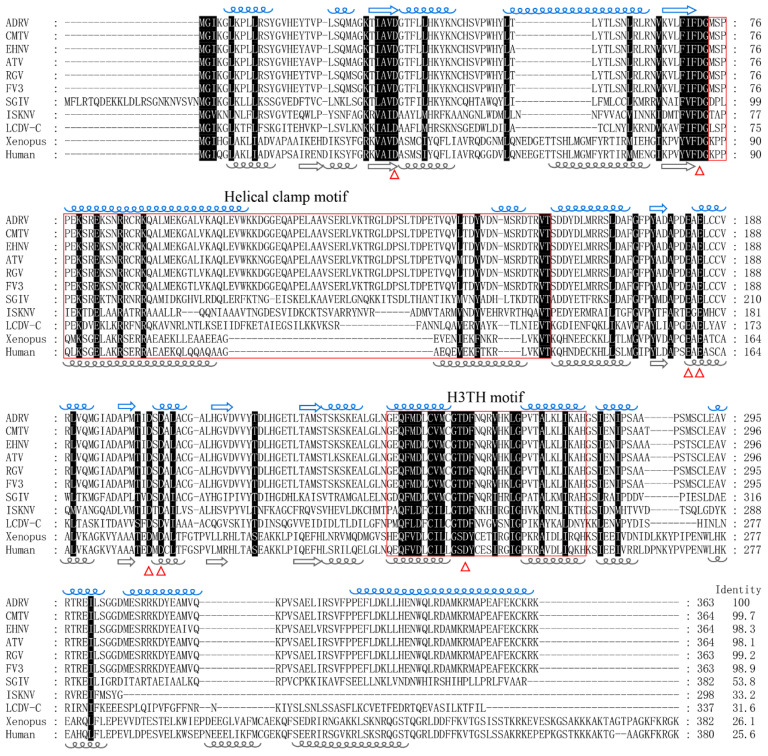
Multiple amino acid sequence alignment of ADRV *12L* with its homologs in iridoviruses and FEN1 of *Xenopus tropicalis* and human. CMTV, common midwife toad virus; EHNV, epizootic hematopoietic necrosis virus; ATV, *Ambystoma tigrinum* virus; RGV, *Rana grylio* virus; FV3, frog virus 3; SGIV, Singapore grouper iridovirus; ISKNV, infection spleen and kidney necrosis virus; LCDV-C: lymphocystis disease virus isolated from China. The GenBank accession numbers of these proteins are shown in Table S2. The black shaded regions indicate completely conserved residues. The conserved putative active sites (aspartic acid, D; glutamic acid, E) are marked with triangles at the bottom. The predicted α-helices (blue helical lines) and β-sheets (blue arrows) of ADRV *12L* are indicated above the sequence. The α-helices (gray helical lines) and β-sheets (gray arrows) of human FEN1 are indicated below the sequence. The predicted helical clamp motif and H3TH motif are indicated with red boxes. The sequence identity between ADRV *12L* and other proteins is shown at the end.

Sequence analysis showed that the ORF of ADRV *12L* was 1092 bp and encoded a peptide of 363 aa with a predicted molecular weight of 40.6 kDa. The ADRV *12L* had homologs in all the iridoviruses sequenced to date. Sequence alignment showed that it had the highest aa sequence identity (99.7%) with its homolog from common midwife toad ranavirus (CMTV) (Figure 1). The lowest sequence identity (31.6%) in iridoviruses was obtained between it and lymphocystis disease virus isolated in China (LCDV-C). The aa sequences of ADRV *12L* and FEN1 of *Xenopus tropicalis* and human were also aligned, which showed an identity more than 25%.

A conserved domain search with the NCBI Conserved Domain Database showed that the domains from the Rad2/FEN1/XPG superfamily were hit in ADRV *12L*. In the sequence alignment shown in Figure 1, the putative enzyme active site, the aspartic acid (D) and glutamic acid (E), were conserved from ADRV *12L* to human FEN1. We further predicted the secondary and three-dimensional structures of ADRV *12L* (Figure S1). The results show that ADRV *12L* contains 16 α-helices and 6 β-sheets. The distribution of α-helices and β-sheets between ADRV *12L* and human FEN1 are highly similar, especially in the region of amino acids 22 to 285 of ADRV *12L*, which contained these conserved active sites (Figure 1). The functional enzyme motif such as the helical clamp motif and H3TH motif were also revealed in the region. These results all indicate that ADRV *12L* is a homolog of the Rad2 family.

### 3.2. Temporal Expression Pattern and Subcellular Localization of ADRV 12L during Infection

**Figure 2:**
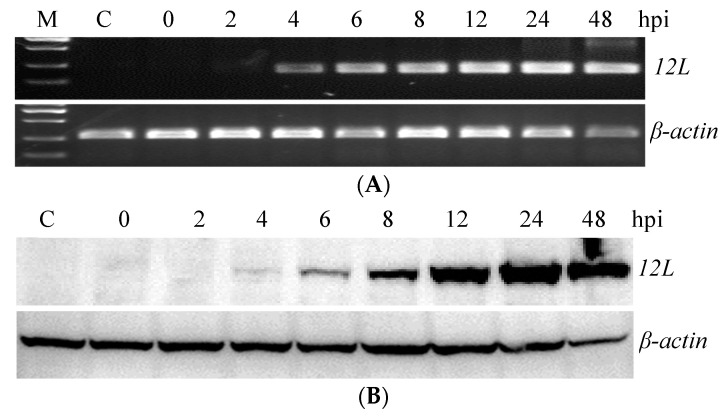

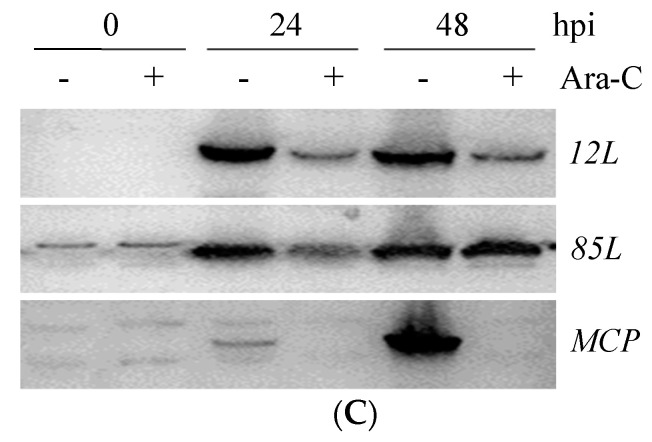
Temporal expression pattern of the *12L* gene and protein in ADRV infected cells. (**A**,**B**) Mock or ADRV infected cells were collected at the indicated time points and analyzed by RT-PCR (**A**) and Western blotting (**B**), respectively. (**C**) Western blot analysis of ADRV *12L* expression in the presence or absence of Ara-C. Detection of 85L and MCP expression was used as control.

RT-PCR showed that the transcription of the ADRV *12L* gene was obvious at 4 hpi. A weak band corresponding to ADRV *12L* transcription was even detected at 2 hpi (Figure 2A). Western blot analysis showed that ADRV *12L* was detected at 4 hpi and its expression increased with the infection time until 24 hpi (Figure 2B). In the Ara-C treatment assay, the band for ADRV *12L* was not detected at 0 hpi but was detected in all the samples at 24 and 48 hpi, although the band in the Ara-C treatment group was weaker than that from the no-drug group (Figure 2C). As a control, the ADRV 85L, which is a single-stranded DNA-binding protein involved in viral DNA replication, was also detected in the presence of Ara-C. The major capsid protein (MCP), whose encoding gene is a late expression gene, was not detected in the presence of Ara-C. Collectively, the results indicated that ADRV *12L* belongs to the early expression class of genes.

**Figure 3:**
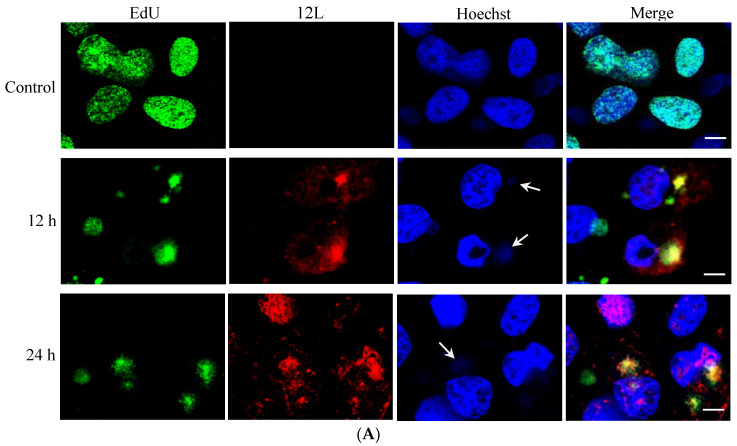

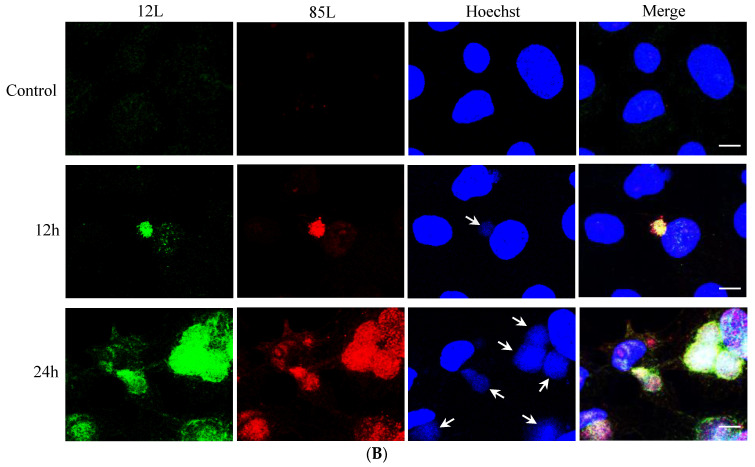
Analysis of the co-localization between ADRV *12L* and nascent DNA or ADRV 85L. (**A**) Mock or ADRV infected cells were labeled with EdU at indicated time points, and then serially stained with Alexa Fluor 488 azide, anti-12L antibody, Alexa Fluor 546 conjugated goat anti-mouse IgG, and Hoechst 33342. EdU labeled nascent DNA was presented in green color. ADRV *12L* was presented in red color. (**B**) Mock or ADRV infected cells were fixed and serially stained with anti-12L antibody (mouse), anti-85L antibody (Rabbit), Alexa Fluor 488 conjugated goat anti-mouse IgG, Alexa Fluor 546 conjugated goat anti-Rabbit IgG, and Hoechst 33342. ADRV *12L* was presented in green color and 85L in red color. The visible Hoechst-stained viral factories were indicated by white arrows. Bar = 10 μm.

The localization of ADRV *12L* in virus infection was further investigated by immunofluorescence. EdU can be used as a substrate in DNA replication. Here, it was used to label the nascent DNA. As shown in Figure 3A, the cell nuclei were labeled with EdU (green color) in the cells without virus infection. In the cells at 12 hpi, the EdU labeled DNA was located in the cytoplasm, where the viral factories are located. ADRV *12L* (red color) could be detected in the whole cell, while it was colocalized with most of the EdU labeled nascent DNA. The localization of ADRV *12L* at 24 hpi was similar to that at 12 hpi. The colocalization of ADRV *12L* and ADRV 85L, which is a single-stranded DNA binding protein involved in replication, was also investigated. The results showed that the two proteins colocalized during virus infection (Figure 3B).

### 3.3. Construction of 12L Deleted Recombinant Virus

**Figure 4:**
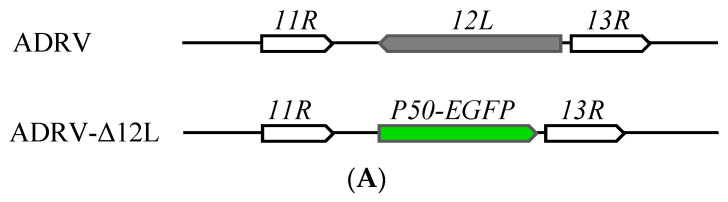

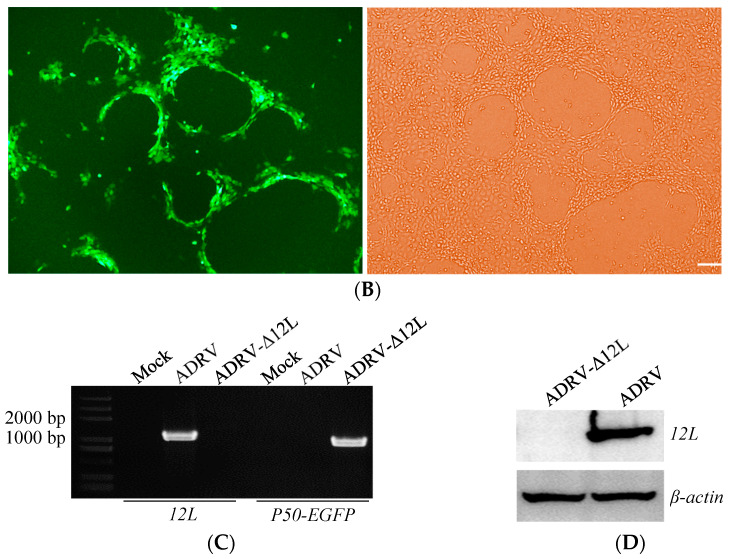
Construction of *12L* deleted recombinant virus ADRV-Δ12L. (**A**) Schematic diagram of ADRV-Δ12L structure. The EGFP gene driven by the virus P50 promoter replaced the coding region of *12L*. (**B**) Light and fluorescence micrographs of ADRV-Δ12L infected cells. The green color overlapped with viral plaques. (**C**) PCR analysis using primers for *12L* and *P50-EGFP*. The *12L* was only detected in wild type ADRV and *P50-EGFP* was only detected in ADRV-Δ12L. (**D**) Western blot analysis of ADRV-Δ12L and ADRV infected cells. The *12L* band was not detected in ADRV-Δ12L infected cells. β-actin was used as an internal control.

A mutant ADRV with deleted *12L* gene (ADRV-Δ12L) was constructed to investigate the role of *12L* in virus infection. In the genome of ADRV-Δ12L, the P50 promoter driving EGFP [28] in a opposite direction replaced the ORF encoding *12L* (Figure 4A), which made the recombinant virus able to be isolated and purified via the green fluorescence protein. The cells infected with ADRV-Δ12L showed plaques and green fluorescence, which completely overlapped (Figure 4B). PCR analysis and Western blotting were further employed to confirm the deletion of *12L*. In wild type ADRV infected cells, the coding sequence for *12L* and a protein band corresponding to *12L* were detected, while no band for *12L* was detected in ADRV-Δ12L infected cells (Figure 4C,D). In ADRV-Δ12L infected cells, the coding sequence for *P50-EGFP* was detected (Figure 4C). These results indicate that the *12L* deleted recombinant virus ADRV-Δ12L was obtained successfully.

### 3.4. Deletion of 12L Impaired DNA Homologous Recombination and Double-Stranded Break Repair

**Figure 5:**
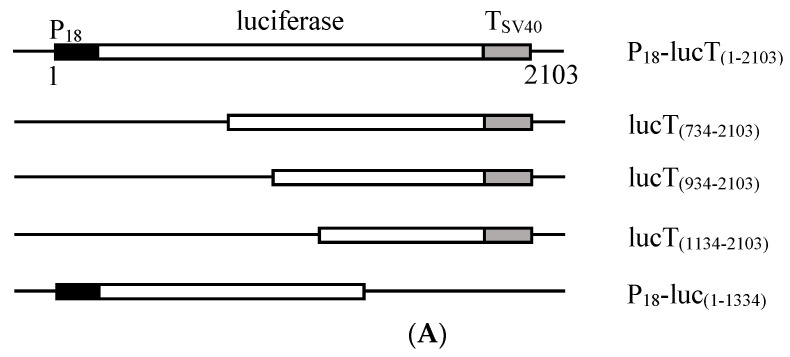

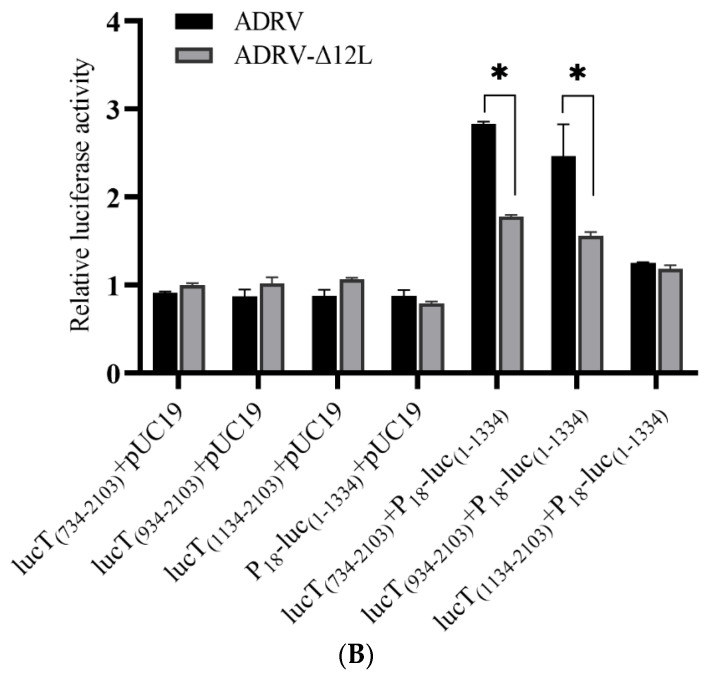
Luc-HR assay. (**A**) Schematic diagram of the viral P18 promoter driving plasmids. The plasmid P_18_-lucT_(1–2103)_ containing the full length of the P18 promoter, firefly luciferase gene, and SV40 terminator has a size of 2103 bp. The other plasmids were constructed based on the plasmid, and their contained regions are shown in parentheses. (**B**) Relative luciferase activity. The cells were infected with ADRV or ADRV-Δ12L for 6 h, and then transfected with different plasmid combinations, respectively. A plasmid containing P18 driving *Renilla* luciferase was transfected simultaneously as an internal control. The detected firefly luciferase activity was normalized to the *Renilla* luciferase activity in each group. In the present figure, the firefly luciferase activity in the ADRV-Δ12L infected and lucT_(734–2103)_ + pUC19 transfected group was set as 1. Experiments were conducted in triplicate and analyzed using Student’s t-test. Significant differences are marked with * (*p* < 0.05).

To evaluate the effect of *12L* deletion on DNA homologous recombination and repair, the Luc-HR assay and DSBR assay were performed. We first constructed a plasmid, P_18_-lucT_(1–2103)_, in which the firefly luciferase gene was promoted by the viral *ICP18* promoter and terminated by the SV40 terminator (Figure 5A). Then, plasmids with different lengths of the P_18_-lucT_(1–2103)_ were constructed. The plasmid, P_18_-luc_(1–1334)_, containing the N-terminal 1334 nt of the P_18_-lucT_(1–2103)_ was co-transfected with plasmids containing different lengths of the C-terminal of P_18_-lucT_(1–2103)_, respectively. 

If the DNA homologous recombination occurred, a full length P_18_ driving luciferase gene would be generated, which would lead to the expression of the firefly luciferase. As shown in Figure 5B, the relative firefly luciferase activity generated in the lucT_(734–2103)_ + pUC19, lucT_(934–2103)_ + pUC19, lucT_(1134–2103)_ + pUC19, and P_18_-luc_(1–1334)_ + pUC19 groups that contained part of the full length P_18_-lucT_(1–2103)_ was low and showed no significant differences between the ADRV and ADRV-Δ12L infected groups. However, in the lucT_(734–2103)_ + P_18_-luc_(1–1334)_ and lucT_(934–2103)_ + P_18_-luc_(1–1334)_ groups, the firefly luciferase activity induced by ADRV was significantly higher than that induced by ADRV-Δ12L. Among them, the firefly luciferase activity of the lucT_(734–2103)_ + P_18_-luc_(1–1334)_ group was higher than that of the lucT_(934–2103)_ + P_18_-luc_(1–1334)_ group. Although the firefly luciferase activity of the lucT_(1134–2103)_ + P_18_-luc_(1–1334)_ group was higher than that of the groups only containing a part of the luciferase gene, no significant difference was observed between ADRV and ADRV-Δ12L.

**Figure 6:**
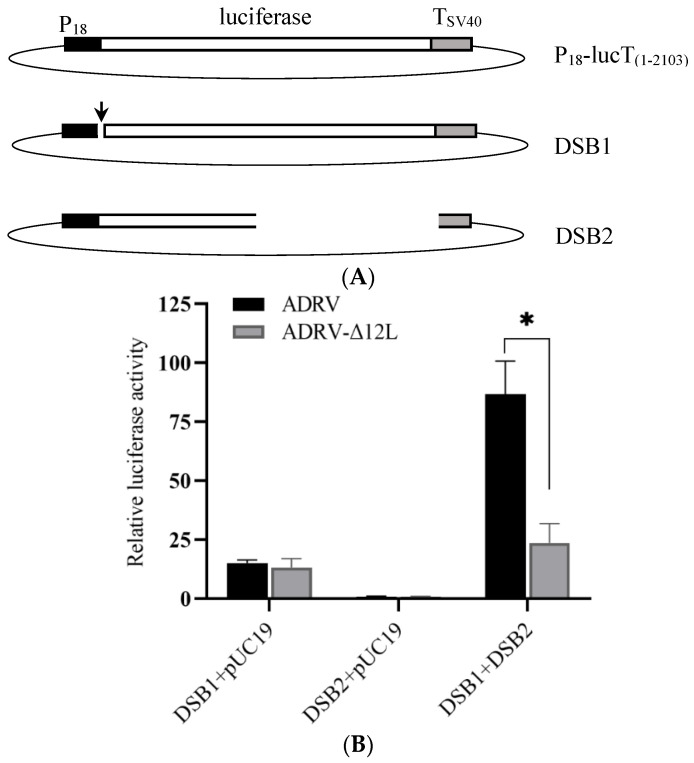
Luciferase-based DSBR assay. (**A**) Schematic diagram of the viral P18 promoter driving plasmids and other constructs. DSB1 had a nick (indicated by a black arrow) between P18 and the ATG of the firefly luciferase gene. DSB2 lacked the C-terminal of the firefly luciferase gene. (**B**) Relative luciferase activity. The cells were infected with ADRV or ADRV-Δ12L for 6 h, and then transfected with different DNA combinations, respectively. A plasmid containing P18 driving *Renilla* luciferase was transfected simultaneously as an internal control. The detected firefly luciferase activity was normalized to the *Renilla* luciferase activity in each group. In the present figure, the firefly luciferase activity in the ADRV infected and DSB2 + pUC19 transfected group was set as 1. Experiments were conducted in triplicate and analyzed using Student’s *t*-test. Significant differences are marked with * (*p* < 0.05).

The DSBR assay was performed using two linearized plasmids: DSB1, which had a nick between the P_18_ promoter and the ATG codon of the firefly luciferase gene, and DSB2, which lacked the C-terminal of the firefly luciferase gene (Figure 6A). The plasmids DSB1 and DSB2 were co-transfected or transfected alone into virus infected cells. If DSBR occurred, the nick would be repaired and firefly luciferase would be generated. As shown in Figure 6B, there was no significant difference between ADRV and ADRV-Δ12L in the DSB1 + pUC19 and DSB2 + pUC19 groups. However, the firefly luciferase activity induced by ADRV was significantly higher than that induced by ADRV-Δ12L in the DSB1 + DSB2 group. In addition, the luciferase activity of the DSB1 + pUC19 group was higher than that of the DSB2 + pUC19 group. Collectively, the results indicate that ADRV caused more HR and DSBR than ADRV-Δ12L.

### 3.5. Deletion of 12L Impaired ADRV Infection

**Figure 7:**
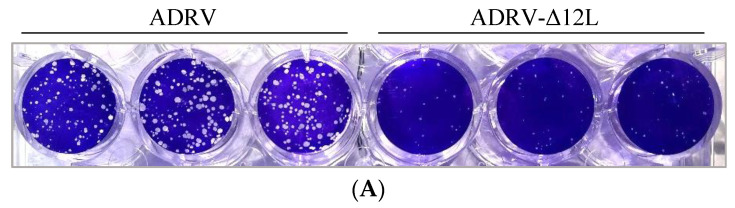

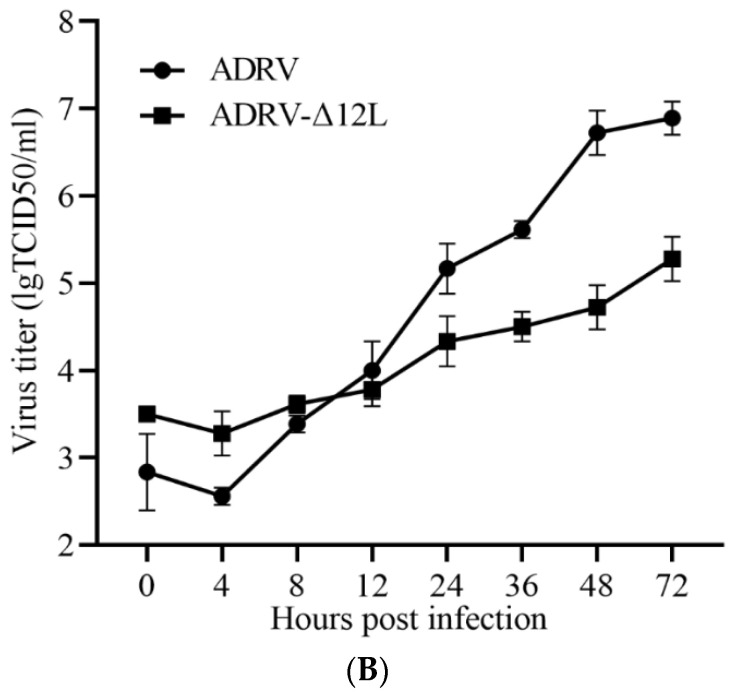
Virus infection was impaired with the deletion of *12L*. (**A**) Plaque assay of ADRV and ADRV-Δ12L. (**B**) One-step growth curves of ADRV and ADRV-Δ12L in GSTC cells. Cells were infected with ADRV or ADRV-Δ12L at an MOI of 1 and then harvested at different times for titration. The average titers of three independent experiments are shown as logTCID50 ± SD.

Plaque assay was performed and one-step growth curves were generated to investigate the role of *12L* in ADRV infection. In the plaque assay, the number of plaques induced by wild type ADRV was more than that in ADRV-Δ12L infected cells. Meanwhile, the size of the plaque in ADRV infected cells was larger than that in ADRV-Δ12L infected cells (Figure 7A).

In the one-step growth curves, the titers of ADRV and ADRV-Δ12L all increased from 4 hpi, but the titers of ADRV increased more rapidly than those of ADRV-Δ12L. From 12 hpi, the ADRV titers were significantly higher than the ADRV-Δ12L titers (Figure 7B). These results suggest that the infection efficiency of ADRV was impaired by the absence of the *12L* protein.

## 4. Discussion

ADRV *12L* and its homologs (such as 95R of frog virus 3, and 102R of *Rana grylio* virus) have been considered as core genes of iridoviruses and were predicted as Rad2 family proteins, but their functions had not been investigated. In the present study, we cloned and characterized ADRV *12L* as a virus encoded protein involved in DNA homologous recombination and repair, and it had been identified as a protein associated with viral nascent DNA in our previous study on the ranavirus replisome and transcription complex [20]. 

Iridoviruses have been shown to possess a complex gene regulation strategy in which genes are expressed in three temporal kinetic stages: immediate early, early, and late [1,26]. The early genes are expressed before virus DNA replication and the late genes are expressed after the onset of virus DNA replication [26]. Therefore, the early and late genes can be discriminated by the use of DNA replication inhibitor. In the present study, the expression of ADRV *12L* was inhibited by the addition of Ara-C, indicating that ADRV *12L* is an early expression gene. The FV3 *95R* that encodes the homolog of ADRV *12L* has been identified as an early gene [17]. Virus early genes usually encode proteins involved in virus–host interactions, virus DNA replication, and transcription. 

EdU (5-ethynyl-2′-deoxyuridine) is an analog of deoxythymidine and has been used for DNA labeling with copper-catalyzed azide-alkyne cycloaddition reactions [30]. It has also been used for tracking viral genomes in host cells [31]. As EdU is used as a substrate during DNA synthesis in replication, EdU-labeled DNA is the nascent DNA. In the localization assays performed in the present study, most of the EdU-labeled viral DNA was colocalized with the ADRV *12L* protein, indicating that ADRV *12L* participated in the DNA replication process. However, still some EdU labeled loci did not colocalized with ADRV *12L*, suggesting that ADRV *12L* may be absent in some steps during the DNA synthesis. Another colocalization assay conducted with ADRV 85L, which is a viral single-stranded binding protein, produced similar results.

To further investigate the possible function of ADRV *12L* in virus replication, we obtained a *12L* deleted mutant ADRV. The ADRV-Δ12L verified that *12L* could be deleted from the ADRV genome, but its deletion affected the plaque formation and virus titer. This phenomenon was also observed in a mutant vaccinia virus with the FEN1 homolog deleted [16].

Homologous recombination comprises a series of pathways that function in DNA repair and is also critical for DNA replication [32]. Plasmid-based recombination assays have been used in research on the enzymes involved in poxvirus DNA repair and recombination [16,29], in which a poxvirus promoter driving luciferase was detected and β-galactosidase was used as an internal control. In the present study, we modified the system by using the immediate-early promoter (P18) from ADRV to drive the firefly luciferase, which caused the firefly luciferase gene to be expressed immediately after a full ORF was generated. During the temporal cascade expression of iridovirus genes, the expression of late genes is regulated by genome replication. Therefore, other promoters, such as the promoter from the virus late gene, will be largely affected by the efficiency of virus genome replication, which could be influenced by the deletion of *12L*. The P18 promoter was used to minimize the effect of the deletion of *12L* on the efficiency of gene expression. In addition, different from the β-galactosidase used as the internal control in the previous study [16], we used the *Renilla* luciferase, which was also driven by the viral immediate-early promoter, as the internal control, for the convenience of using the detection kit. 

In the preliminary experiments, we also estimated the possible effect of the viral doses of ADRV and ADRV-Δ12L on their inductions of luciferases. The plasmid P_18_-lucT_(1–2103)_ containing the full length of firefly luciferase was transfected into the two virus infected groups of cells (at the same dose of 0.5 MOI). The results showed that there were no significant differences in luciferase activity between ADRV and ADRV-Δ12L infected groups when the same viral dose was used, which indicated that the viral doses used in the assay were appropriate and also verified the suitability of using the viral IE promoter. The relative luciferase activity in the ADRV-Δ12L infected group was lower than that in the ADRV infected group in both of the Luc-HR and DSBR assays, indicating that *12L* participated in these reactions. However, increased firefly luciferase was still observed in the ADRV-Δ12L infected group, indicating that the HR or DSBR still occurred under 12L-lacking conditions. It suggested that there were other proteins possessing related enzyme activities. It has been reported that the DNA polymerase of vaccinia virus has a role in virus genetic recombination [33]. Whether the DNA polymerase of ADRV has similar activity needs further research. In addition, the ADRV 50L was predicted to contain motifs from Holliday junction resolvases, which could be involved in DNA repair [8,34]. 

In the Luc-HR assay, plasmids with different length of overlap regions were designed to make combinations. The HR process includes steps, such as homology search, strand invasion, and DNA synthesis [35,36]. Long overlap regions could be beneficial for the homology search and strand invasion steps. As expected, we observed that the plasmid combinations with longer overlap regions induced higher firefly luciferase activities than those plasmids with smaller overlap regions. The phenomenon also verified the occurrence of HR in these plasmid combinations. The HR-based gene knockout has been used in the research of gene functions of ranaviruses [28,37,38]. Our results suggest that overlap regions (homologous arms) with appropriate lengths were needed in the design of the HR-based gene knockout assays in ranaviruses.

There are two major pathways for the repair of DSBs in eukaryotic cells: HR and nonhomologous DNA end joining (NHEJ) [39]. The repair of DSBs by HR involves DNA resection, homology search, strand invasion, DNA synthesis, etc. [35]. The NHEJ pathway is mainly mediated by proteins including nucleases, polymerases, and ligase complexes [40]. In the DSBR assay performed in the present study, although the firefly luciferase activity between ADRV and ADRV-Δ12 infected samples of the “DSB1 + con” and “DSB2 + con” groups was similar, the activity in the “DSB1 + con” group was higher than that in the “DSB2 + con” group. It seems like the firefly luciferase gene was expressed in the “DSB1 + con” group, which hinted that other proteins possessing ligation functions could repair the nick between P18 and the ATG in DSB1.

Two aquatic animal cell lines were used in the present study to investigate the roles of ADRV *12L*. The GSTC cell line was derived from the thymus tissue of Chinese giant salamander (*A. davidianus*) [21], which is the natural host of ADRV. The EPC cell line has been widely used in virological investigations in aquatic animals, including ranaviral research [22,41]. Investigation with the two cell lines revealed the important function of ADRV *12L* in ADRV infection in vitro. However, whether it has the same function in virus infection in vivo needs to be proved with appropriate animals in the future.

In conclusion, the present study with the knockout mutant virus and luciferase-based HR and DSBR assays confirmed that the Rad2 homolog ADRV *12L*, which is conserved in iridoviruses, plays important roles in DNA recombination and repair, and, thus, is important for virus efficient infection. This work also contributes to the further understanding of ranavirus replication.

## Data Availability

Not applicable.

## Supplementary Materials

The following supporting information can be downloaded at: https://www.mdpi.com/article/10.3390/v14050908/s1. Figure S1: Predicted structure of ADRV *12L*; Table S1: Primers used in the study; Table S2: GenBank accession numbers of the Rad2 homologs used in the sequence alignment.
