# Supplementary material for: ADRV 12L: A Ranaviral Putative Rad2 Family Protein Involved in DNA Recombination and Repair
Source: Viruses. 2022 Apr 27;14(5):908. doi: 10.3390/v14050908 (PMC9146916; doi:10.3390/v14050908)
Supplement: Supplementary file 1 [file viruses-14-00908-s001.zip › Fig S1 Predicted structure of ADRV 12L.pdf]

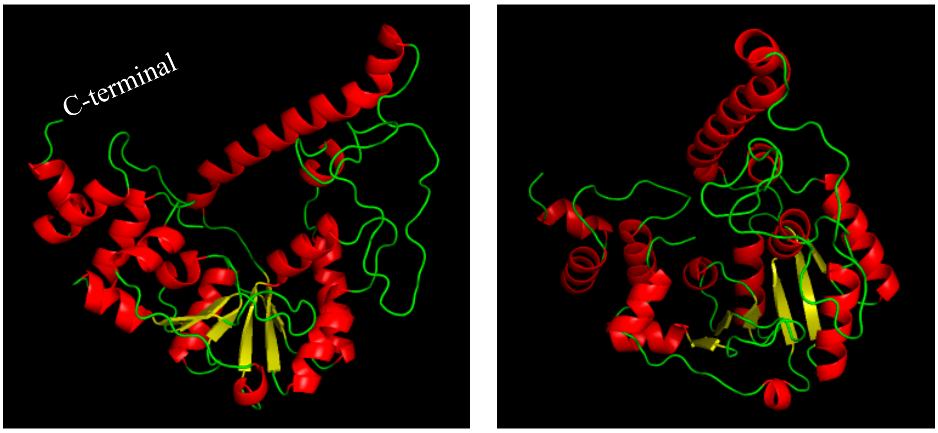

Figure S1 Predicted structure of ADRV 12L. The structure was predicted by RoseTTAFold in the Robetta (http://robetta.bakerlab.org/) and visualized by PyMOL. The two figures were obtained from different directions. Red, Helix; yellow, sheet; green, loop.
