# Supplementary material for: ADRV 12L: A Ranaviral Putative Rad2 Family Protein Involved in DNA Recombination and Repair
Source: Viruses. 2022 Apr 27;14(5):908. doi: 10.3390/v14050908 (PMC9146916; doi:10.3390/v14050908)
Supplement: Supplementary file 1 [file viruses-14-00908-s001.zip › table S1 primers.pdf]

Table S1 Primers used in the study.

Primers	Sequences (5'-3')	Constructs or applications
12L-F	GTGCGGCACAGACTTTAACC	RT-PCR
12L-R	ATGTCCCCGCCAGAGAGTAT	
β-actin-F	CCACTGCTGCCTCCTCTT	
β-actin-R	GCAATGCCTGGGTACATG	
32a-12L-F	ACAAGAATTCTCTCCACCCGAAAAGTCTAG	Protein expression
32a-12L-R	TACTAAGCTTTCATCCGAGGGCCTCCTTGG	
$12L_L$-F	GCCAAGACCAAGGACACACTGTCC	pMD18T-$12L_{-L}$-GFP-P50-$12L_{-R}$ used in mutant virus construction
$12L_L$-EGFP-R	CTTGCGCTTTCTCAGAGGTTTTTGGTTGTTTAACTCGTAGG	
$12L_L$-EGFP-F	CCTACGAGTTAAACAACCAAAAACCTCTGAGAAAGCGCAAG	
EGFP-$12L_R$-R	CTCGTACTACACACTCCCACAACTAGAATGCAG	
EGFP-$12L_R$-F	CTGCATTCTAGTTGTGGGAGTGTGTAGTACGAG	
$12L_R$-R	CAGAAAGTTTGCAGAGCGTCAGCTC	
R12L-F	ATGGGCATAAAAGGACTGAAACCCCTTC	Detection of *12L*
R12L-R	TCACTTGCGCTTGCACTTCTCAAAGG	
P50-EGFP-F	AACCTCTGAGAAAGCGCAAGCCTC	Detection of *P50-EGFP*
P50-EGFP-R	TTACTTGTACAGCTCGTCCATGCC	
P18-luci-40-F	ACTAGGTCCGCCGACGAGCCCATG	P_{18}-$lucT_{(1-2103)}$
P18-luci-F	ACACTTTACATTCACAATGCGCATGGAAGACGCCAAAAACATAAAG	
P18-luci-R	CTTTATGTTTTTGGCGTCTTCCATGCGCATTGTGAATGTAAAGTGT	
luci-40-F	GGAAAGATCGCCGTGTAAAGCGGCCGCGACTCTAGATC	
luci-40-R	GATCTAGAGTCGCGGCCGCTTTACACGGCGATCTTTCC	
P18-luci-40-R	TAAGATACATTGATGAGTTTGGAC	
P18-Rluc-F	ACATTCACCATGAGCATGACTTCGAAAGTTTATGATCC	P_{18}-Rluc
P18-Rluc-R	GGATCATAAACTTTCGAAGTCATGCTCATGGTGAATGT	
Rluc-R	TACCACATTTGTAGAGGTTTTACTTG	
18luci40-1334R	CAGATCCACAACCTTCGCTTC	P_{18}-$luc_{(1-1334)}$
18luci40-734F	AATGAATACGATTTTGTGCCAG	$lucT_{(734-2103)}$
18luci40-934F	CCATCACGGTTTTGGAATGTTTAC	$lucT_{(934-2103)}$
18luci40-1134F	ACGAAATTGCTTCTGGTGGC	$lucT_{(1134-2103)}$
DSB-F1	ATGGAAGACGCCAAAAACATAAAGAAAG	DSB1
DSB-R1	GCTCATGGTGAATGTAAAGTGTTTTAG	
DSB-F2	GCCGCGACTCTAGATCATAATC	DSB2
DSB-R2	GTAAACATTCCAAAACCGTGATGG	
