# Supplementary material for: ADRV 12L: A Ranaviral Putative Rad2 Family Protein Involved in DNA Recombination and Repair
Source: Viruses. 2022 Apr 27;14(5):908. doi: 10.3390/v14050908 (PMC9146916; doi:10.3390/v14050908)
Supplement: Supplementary file 1 [file viruses-14-00908-s001.zip › table S2 accession numbers.pdf]

Table 2 GenBank accession numbers of the Rad2 homologues used in the sequence alignment.

Virus	Protein	Accession number
Andrias davidianus ranavirus (ADRV)	12L	AGV20543.1
Frog virus 3 (FV3)	95R	YP_031674.1
Rana grylio virus (RGV)	102R	AFG73144.1
Common midwife toad virus (CMTV)	12L	ASH98834.1
Epizootic haematopoietic necrosis virus (EHNV)	10L	YP_009182009.1
Ambystoma tigrinum virus (ATV)	9L	ALN37112.1
Singapore grouper iridovirus (SGIV)	97L	YP_164192.1
Lymphocystis disease virus isolated in China (LCDV-C)	169R	YP_073674.1
Infectious spleen and kidney necrosis virus (ISKNV)	27L	NP_612249.1
Xenopus tropicalis	flap endonuclease 1	NP_001017005.1
Homo sapiens	flap endonuclease 1	NP_004102.1
